# Prevalence of depression and its associated factors among patients attending primary care settings in the post-conflict Northern Province in Sri Lanka: a cross-sectional study

**DOI:** 10.1186/1471-244X-14-85

**Published:** 2014-03-24

**Authors:** Upul Senarath, Kolitha Wickramage, Sharika Lasanthi Peiris

**Affiliations:** 1 Department of Community Medicine, Faculty of Medicine, University of Colombo, 25 Kynsey Road, Colombo 08, Sri Lanka; 2 International Organization for Migration Sri Lanka, No. 62, Green Path, Ananda Coomaraswamy Mawatha, Colombo 03, Sri Lanka

**Keywords:** Depression, Mental health, Primary care, Post-conflict, Patient Health Questionnaire, PHQ-9

## Abstract

**Background:**

In Sri Lanka, civilians in the Northern Province were affected by a long-term armed conflict that ended in 2009. This study aims to describe the prevalence of depression and its associated factors among adult patients attending primary care settings in the Northern Province in Sri Lanka.

**Methods:**

We report data from a cross-sectional patient morbidity registry established in 16 primary care facilities (12 Divisional Hospitals and 4 Primary Medical Care Units) in four districts of the Northern Province. The Patient Health Questionnaire-9 (PHQ-9) was used to assess depression among all patients aged ≥18 years, between March and May 2013. A sample of 12,841 patient records was included in the analysis. A total score of ≥10 in the PHQ-9 was considered as major depression. Factors associated with major depression were tested using multivariable logistic regression analysis.

**Results:**

The prevalence of major depression was 4.5% (95% CI: 4.1-4.9) and mild depression was 13.3% (95% CI: 12.7-13.9). The major depression was significantly higher in females than males (5.1% vs. 3.6%) and among unpaid family workers (6.0%) than any other category who earned an income (varied between 1.2% and 3.2%). The prevalence was rising significantly with advancing age, and ranged from 0.3% in the youngest to 11.6% in the elderly.

Multivariable regression analysis revealed that the females have a higher risk for major depression than males (OR = 1.4; 95% CI: 1.1-1.7). Older patients were more likely to be depressed than younger patients, OR (95% CI) were 4.9 (1.9-12.5), 5.6 (2.2-14.0), 5.7 (2.3-14.2) and 4.7 (1.8-11.9) for the age groups 25–34, 35–49, 50–64, and ≥65 years respectively, in contrast to 18–24 year group. Disability in walking (OR = 7.5; 95% CI: 5.8-9.8), cognition (OR = 4.5; 95% CI: 3.6-5.6), self-care (OR = 2.6; 95% CI: 1.7-4.0), seeing (OR = 2.3; 95% CI: 1.8-3.0), and hearing (OR = 2.0; 95% CI: 1.5-2.5) showed significant associations with depression.

**Conclusions:**

Depression is a common issue at primary care settings in a post-conflict population, and the elders, women and persons with disability are at a greater risk. Strengthening capacity of primary care facilities and community mental health services is necessary for early detection and management.

## Background

Depression is a major public health problem that affects patients and society [1,2]. The clinical condition named as unipolar depression, is characterized by depressed mood, hopelessness, helplessness, intense feelings of guilt, sadness, low self-esteem, thoughts of self-harm and suicide [3]. The estimates of global disease burden ranked depression as the fourth leading cause of disease burden in the year 2000, accounting for 4.4% of total Disability Adjusted Life Years (DALYs) [4]. Depression is known as one of the most prevalent yet treatable mental disorders presenting in general medical as well as specialty settings [5,6]. About one in ten patients seen in the primary care settings suffers from some form of depression [7,8]. According to a systematic review, depression substantially increases the risk of death, and gives rise to other chronic disease conditions such as cardiovascular disease [9].

The World Mental Health Survey reported that 15% of the population from high-income countries compared to 11% from low and middle-income countries were likely to get depression over their lifetime [10]. Globally, 5.5% reported having an episode of depression in the previous year. Studies from the South Asian region show varying prevalence of depression possibly due to differences in the study settings and instruments used to measure it. For example, the age-adjusted prevalence of depression in an urban South Indian population was 15.9% according to *Patient Health Questionnaire-12* (PHQ-12) [11], in contrast to 45.9% in urban Pakistan according to *depressive symptom questionnaire*[12]. It was found to be 29% in rural Bangladesh using *the Montogomery and Aasberg Depression Rating Scale (MADRS)*[13]. In Sri Lanka, there is a scarcity in mental health research, and only few studies have reported depression and its correlates [14]. According to the national mental health survey of Sri Lanka conducted in 2007, depression was assessed using the *Patient Health Questionnaire-9* (PHQ-9), and the prevalence of major depression was 2.6% [15].

The effects of war on mental health have been documented in previous literature, and the common war-related mental health conditions included post-traumatic stress disorder (PTSD), anxiety and depression [16-18]. Studies in the post-conflict populations showed a definite increase in the incidence and prevalence of mental disorders, with women being more affected than men. Prevalence rates were associated with the degree of trauma, and the availability of physical and emotional support [19]. A study in Sudan after 20 years of war reported a high prevalence of mental disorders, for example, over one third of respondents met symptom criteria for PTSD, and half of respondents met symptom criteria for depression [20].

In Sri Lanka, civilians in the Northern Province were affected by 30-year long armed conflict that ended in May 2009. A household survey in 2009 among residents of Jaffna district in the Northern Province revealed a substantially high prevalence of symptoms of war-related mental health conditions, which were significantly associated with displacement status and underlying trauma exposure. In this survey, the overall prevalence of PTSD, anxiety, and depression were 7.0%, 32.6% and 22.2% respectively [21]. A high prevalence of common mental disorders (18.8%) was reported among internally displaced persons, particularly where displacement was prolonged, with major depression at 5.1% and other depressive syndromes at 7.3% [22]. A study in school children in areas affected by the armed conflict reported that the great majority of children experienced severely traumatizing events such as combat, bombing, shelling, or witnessing the death of a loved one, and their performance and functioning were related to the total load of traumatic events experienced [23]. According to a recent qualitative study in Northern Sri Lanka, complex mental health and psychosocial problems at the individual, family and community levels in a post-war context were found to impair recovery [24]. These studies indicate that more efforts related to psychological health are needed to re-establish the normalcy in the region despite the re-settlement of the internally displaced persons in the Northern province, together with infrastructure and livelihood development programmes by the state and non-state sectors.

Improving services of primary health care facilities would provide easy access to a wider population for their basic health needs, and reduce overcrowding of larger hospitals. The Primary Medical Care Units and Divisional Hospitals (PMCU/DH) in the Northern Province have been strengthened after the conflict, with the aim of providing first contact care to treat minor illnesses and screen and refer patients for health care institutions at higher level. We assume that the prevalence of depression and other psychosocial issues would be higher in a post-conflict population that seeks care from these facilities in the Northern Province than in the rest of the country. The aim of this paper is to report on the burden of depression and describe the factors associated with depression in a post-conflict population, using a large sample of adult patients accessing primary medical care settings in the Northern Province in Sri Lanka.

## Methods

### Design, setting and participants

A cross-sectional descriptive study was conducted in selected primary health care facilities in the districts of Jaffna, Mannar, Kilinochchi and Mullaitivu. There are 61 Divisional Hospitals (DH) and 35 Primary Medical Care Units (PMCU) in the Northern Province [25], and a rapid rise in the patient influx has been observed in these centres over the recent past possibly due to improvement of health facilities. The study was carried out in 16 purposively-selected primary care centres comprised of 12 DH and 4 PMCU, which collectively provide outpatient care for approximately 2000 patients per week. These sites were purposively selected to capture the health institutions accessed by both recently resettled conflict-affected population and the host population. These 16 health facilities are geographically scattered across the four districts, and serve to the re-settled communities as well as the host populations. A patient morbidity registry was established for the purpose of the study in these centres. Eligible participants included adult men and women aged 18 years or more, who were seeking care at the Out Patient Departments of these study centres. Pregnant women and patients who were severely ill or required emergency hospital admission were excluded. The individuals who had been interviewed once were not included again at their subsequent visits. The data were collected between March and May 2013, and can be considered as a reflection of patients seeking primary care services in the Northern Province due to representation of both resettled and host populations, geographical distribution, and duration of data capture. The sample size was calculated to estimate prevalence of depression at 26% (the highest reported figure in Sri Lanka using the same instrument), with 95% confidence intervals within ±1% precision. The sample size was doubled to account for variation between centres (design effect of 2), and further expanded by 5% to adjust for invalid and incomplete records. The expected sample size was 15,500, and the anticipated time period to cover this sample was 8 weeks according to the average patient load. All eligible participants were recruited consecutively to obtain the required sample size.

### Data collection instrument and methods

We used the Patient Health Questionnaire-9 (PHQ-9) to assess depression. The PHQ-9 is a widely used instrument in primary health care settings, and has the advantage of its exclusive focus on the 9 diagnostic criteria for DSM-IV depressive disorders [26,27]. The PHQ-9 has 9 questions with a score ranging from 0 to 3 for each question. It has been validated and used for screening and diagnostic purposes in different settings both developed and developing countries including Sri Lanka [10,28-30]. Shortness coupled with its construct and criterion validity makes the PHQ-9 an attractive, dual purpose instrument for making diagnoses and assessing severity of depressive disorders, particularly in the busy setting of clinical practice [26]. The PHQ-9 has been previously translated into Sinhalese and Tamil languages and used in the National Mental Health Survey of Sri Lanka conducted in 2007 [29]. The present study used the Tamil version with minor modification to improve its cultural validity.

In addition, a series of six questions was adopted from the questionnaire of Sri Lanka census of population and housing to assess level of disability, which was originally developed by United Nations Washington Group on Disability Statistics [31,32]. The 6-item disability scale used in the present study has been previously validated [31]. These 6 questions on disability can provide a reasonable estimate on people with disabilities, and have been frequently used in population census and surveys for measuring disability prevalence worldwide. The levels of difficulties on seeing, hearing, walking, cognition, self-care and communication were marked on a 3-point rating scale. Data on basic socio demographic data such as age, gender, and nature of occupation of the participants were also included in the morbidity register. All interviews were conducted in Tamil language which is the native language of all participants as well as the interviewers.

The medical officers attached to the selected DH and PMCU were trained adequately to interview participants and record data in the Patient Morbidity Registers. Reliability of data was ensured through several practice interviews during the training. A field coordinator monitored the process of data collection and received the registers through health authority fortnightly for data entry. Validity of the data was also verified at the time of data entry by the field officers.

### Data analysis

The PHQ-9 scores each of the 9 DSM-IV criteria as “0” (not at all) to “3” (nearly every day), with a maximum score of 27. A previous study has reported that PHQ-9 score of 10 and above had a sensitivity of 88% and a specificity of 88% for major depression. PHQ-9 scores of 5, 10, 15, and 20 represented mild, moderate, moderately severe, and severe depression, respectively [33].

In our study, the prevalence major depression was defined as the proportion of individuals with a total score of 10 or more in the PHQ-9. The cutoffs of 5, 10, 15, and 20 represented the thresholds for mild, moderate, moderately severe, and severe depression, respectively. Data on disability were dichotomized as ‘no disability’ vs. ‘any disability’ by combining ‘some difficulty’ and ‘not possible’ categories together. The cross tabulations were generated between prevalence vs. age category, sex, occupation, district and different disabilities. Univariable regression analyses were performed to calculate the odds ratios (OR) and 95% confidence intervals (95% CI) to indicate the magnitude of risk of each independent variable for major depression. In the multivariable analysis, logistic regression models were used in a stepwise backward manner, to calculate adjusted Odds Ratios. All independent variables were entered at the beginning step, while those non-significant were removed in a stepwise manner. The variables retained in the final step were the age, sex, district, and disabilities in seeing, hearing, walking, cognition and self-care. Data were analyzed using SPSS 16.0 evaluation version.

### Ethical considerations

Ethics clearance was granted by the Ethics Review Committee of the Faculty of Medicine, University of Colombo (Reference No. EC-13-066). Informed verbal consent for participation in the study was obtained from participants and the study did not include any person less than 18 years of age. Strict confidentiality and privacy were maintained during interviews and on all personal records. Patients who were found to have any depression were given appropriate medical advice by the medical officers, and those with major depression were referred for necessary action using a referral pathway. This referral mechanism has been already in-place in the mental health programme of the existing health system in the Northern Province.

## Results

### Sample characteristics

Of the total 12,973 participants recruited over a period of 8 weeks, 132 (1.2%) were excluded due to missing data in their records. As shown in Table 1, the analytic sample consisted of 12,841 individuals, with the majority being between the ages 25 and 64 years (77.3%), and women (56.5%). The mean age was 43.2 years with a standard deviation of 15.6. Relatively a small percentage (13%) was formally employed either in government or private sector, almost one-fourth (24.9%) was working on their own account, while the majority (55.5%) was unemployed thus categorized as unpaid workers contributing to family enterprise. The rates of disability, as defined by the proportion having difficulty or inability in performing a particular function, were relatively high for seeing (18.4%), and walking (17.2%) and low for self-care (1.1%) and communication (1.2%).

**Table 1:** Summary of the sample characteristics of respondents (n = 12,841)

**Variable**	**Number**	**(%)**
Age group (years)		
18-24	1475	11.5
25-34	3025	23.6
35-49	3888	30.3
50-64	3019	23.5
65 and above	1434	11.2
Sex		
Male	5578	43.4
Female	7263	56.6
Nature of employment		
Govt./Semi govt. paid employee	1071	8.3
Private sector paid employee	595	4.6
Employer	857	6.7
Own account worker	3197	24.9
Contributing to family enterprise	7121	55.5
District		
Jaffna	4930	38.4
Kilinochchi	3392	26.4
Mannar	509	4.0
Mullaitivu	4010	31.2
Disabilities^a^		
Seeing		
No difficulty	10478	81.6
Difficult or not possible	2363	18.4
Hearing		
No difficulty	11840	92.2
Difficult or not possible	1001	7.8
Walking		
No difficulty	10636	82.8
Difficult or not possible	2205	17.2
Cognition		
No difficulty	11758	91.6
Difficult or not possible	1083	8.4
Self-care		
No difficulty	12697	98.9
Difficult or not possible	144	1.1
Communication		
No difficulty	12687	98.8
Difficult or not possible	154	1.2
Total	**12,841**	**100**

*Govt*. government; *DH* divisional hospital; *PMCU* primary medical care unit.

^a^Those responded difficult or not possible at all are considered as having disability.

### Prevalence of depression

The PHQ-9 in the present sample was found to have a high internal consistency as indicated by Cronbach’s alpha of 0.79. As displayed in Table 2, 13.3% of the respondents had mild depression (PHQ score 5 to 9) and 3.3% moderate depression (PHQ score 10 to 14). Less than 1% were found to have moderately severe (PHQ score 15 to 19) and severe (PHQ score ≥ 20) depression. Overall, the proportion of patients with any depression was 17.8%.

**Table 2:** Prevalence depression as measured by PHQ-9 among patients attending primary health care facilities in the Northern Province (n = 12,841)

**Level of depression**	**%**	**95% CI**		**n**
No depression	82.2	81.5	82.9	10559
Mild depression	13.3	12.7	13.9	1706
Moderate depression	3.6	3.3	3.9	468
Moderately severe depression	0.8	0.6	1.0	101
Severe depression	0.1	0.0	0.2	7
Total	100.0			12841

**Figure 1:**
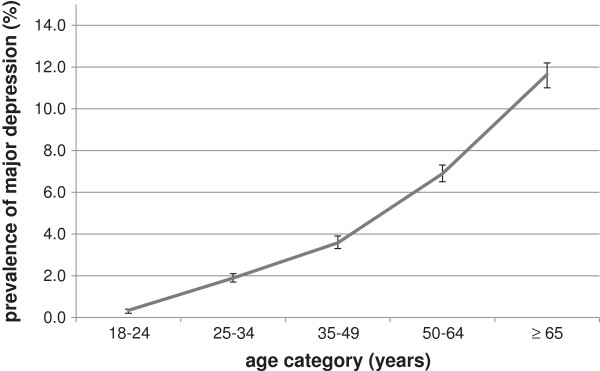
**Trend of major depression according to age category of patients attending primary health care facilities in the Northern Province (n=12,841).** χ^2^ (df=1) for linear trend = 303; p<0.0001. Error bars indicate 95% confidence intervals.

The prevalence of major depression as indicated by the total PHQ score of 10 and above was 4.5% (95% CI: 4.1-4.9) (Table 3). The prevalence of major depression was significantly higher in females than males (5.1% (95% CI: 4.1-5.5) vs. 3.6% (95% CI: 3.3-3.9)) and unpaid family workers (6.0% (95% CI: 5.6-6.4)) than any other category who earned an income (varied between 1.2% (95% CI: 1.0-1.4) and 3.2% (95% CI: 2.9-3.5). As illustrated in Figure 1, the prevalence was rising significantly with advancing age, which ranged from 0.3% (95% CI: 0.2-0.4) in the youngest to 11.6% (95% CI: 11.0-12.2) in the elderly (χ^2^ (df = 1) for linear trend = 303; p < 0.0001). Significantly higher rates of major of depression were reported in all 6 categories of disability, ranging from 16.5% to 43.1%.

**Table 3:** Prevalence depression as measured by PHQ-9 among patients attending primary health care facilities in the Northern Province, according to selected socio demographic characteristics (n = 12,841)

**Variable**	**Major depression %**	**95% CI**		**Total**
PHQ Score	≥10			
Age group (years)^a^				
18-24	0.3	0.2	0.4	1475
25-34	1.9	1.7	2.1	3025
35-49	3.6	3.3	3.9	3888
50-64	6.9	6.5	7.3	3019
65 and above	11.6	11.0	12.2	1434
Sex				
Male	3.6	3.3	3.9	5578
Female	5.1	4.7	5.5	7263
Nature of employment				
Govt./Semi govt. paid employee	1.4	1.2	1.6	1071
Private sector paid employee	3.2	2.9	3.5	595
Employer	1.2	1.0	1.4	857
Own account worker	3.2	2.9	3.5	3197
Contributing to family enterprise	6.0	5.6	6.4	7121
District				
Jaffna	6.5	6.1	6.9	4930
Kilinochchi	2.4	2.1	2.7	3392
Mannar	5.1	4.7	5.5	509
Mullaitivu	3.7	3.4	4.0	4010
Seeing				
No difficulty	1.8	1.6	2.0	10478
Difficult/not possible	16.5	15.9	17.1	2363
Hearing				
No difficulty	3.1	2.8	3.4	11840
Difficult/not possible	20.5	19.8	21.2	1001
Walking				
No difficulty	1.3	1.1	1.5	10636
Difficult/not possible	20.1	19.4	20.8	2205
Cognition				
No difficulty	2.2	1.9	2.5	11758
Difficult/not possible	29.5	28.7	30.3	1083
Self-care				
No difficulty	4.0	3.7	4.3	12697
Difficult/not possible	43.1	42.2	44.0	144
Communication				
No difficulty	4.2	3.9	4.5	12687
Difficult/not possible	27.3	26.5	28.1	154
Total	4.5	4.1	4.9	12841

^a^Chi-square for linear trend of major depression across age groups = 302; p < 0.0001.

### Factors associated with depression

Univariable and multivariable analyses revealed some significant predictors of major depression (Table 4). Females reported a higher risk of having major depression than males (adjusted OR = 1.4 (95% CI: 1.1-1.7)). Older patients were more likely to have depression than younger patients, the adjusted odds ratios were 4.9 (95% CI: 1.9-12.5), 5.6 (95% CI: 2.2-14.0), 5.7 (95% CI: 2.3-14.2) and 4.7 (95% CI:1.8-11.9) for the age groups 25–34, 35–49, 50–64, and ≥65 years respectively, in contrast to 18–24 year group. Although un-employed persons had a higher risk for major depression in the univariable analysis, this factor was non-significant when adjusted for confounding effects in the multivariable analysis. Effect of disability was highly significant in our analysis, expect for those with disability in communication. Patients with disability in walking (adjusted OR = 7.5 (95% CI: 5.8-9.8)), cognition (adjusted OR = 4.5 (95% CI: 3.6-5.6)), self-care (adjusted OR = 2.6 (95% CI: 1.7-4.0)), seeing (adjusted OR = 2.3 (95% CI: 1.8-3.0)), and hearing (adjusted OR = 2.0 (95% CI: 1.5-5.8)) showed significant effects on depression. Significant differences in major depression were evident across districts. In the multivariable analyses, Kilinochchi and Mannar districts had a 1.5 (95% CI: 1.1-2.1) and 4.6 (95% CI: 2.8-7.4) increase in odds respectively, relative to the referent district of Mullaitivu.

**Table 4:** Factors associated with major depression: unadjusted and adjusted odds ratios (n = 12,841)

**Variable**	**Unadjusted**				**Adjusted**^**a**^			
	**OR**	**95% CI**		**P value**	**OR**	**95% CI**		**P value**
Age group (years)								
18-24	1.0				1.0			
25-34	5.6	2.3	14.1	<0.001	4.9	1.9	12.5	0.001
35-49	10.9	4.5	26.7	<0.001	5.6	2.2	14.0	<0.001
50-64	21.8	8.9	52.9	<0.001	5.7	2.3	14.2	<0.001
65 and above	38.8	15.9	94.6	<0.001	4.7	1.8	11.9	0.001
Sex								
Male	1.0				1.0			
Female	1.4	1.2	1.7	<0.001	1.4	1.1	1.7	0.001
Nature of employment								
Govt./Semi govt. paid employee	1.0							
Private sector paid employee	2.3	1.2	4.6	0.016				
Employer	0.8	0.4	1.9	0.653				
Own account worker	2.3	1.4	4.1	0.002				
Contributing to family enterprise	4.5	2.7	7.6	<0.001				
District								
Mullaitivu	1.0				1.0			
Jaffna	1.8	1.5	2.2	<0.001	1.0	0.8	1.2	0.873
Kilinochchi	0.7	0.5	0.9	0.002	1.5	1.1	2.1	0.013
Mannar	1.4	0.9	2.2	0.112	4.6	2.8	7.4	<0.001
Seeing								
No difficulty	1.0				1.0			
Difficult/not possible	10.9	9.1	13.1	<0.001	2.3	1.8	3.0	<0.001
Hearing								
No difficulty	1.0				1.0			
Difficult/not possible	8.0	6.6	9.6	<0.001	2.0	1.5	2.5	<0.001
Walking								
No difficulty	1.0				1.0			
Difficult/not possible	19.9	16.3	24.3	<0.001	7.5	5.8	9.8	<0.001
Cognition								
No difficulty	1.0				1.0			
Difficult/not possible	18.8	15.7	22.6	<0.001	4.5	3.6	5.6	<0.001
Self-care								
No difficulty	1.0				1.0			
Difficult/not possible	17.9	12.7	25.2	<0.001	2.6	1.7	4.0	<0.001
Communication								
No difficulty	1.0							
Difficult/not possible	8.5	5.9	12.3	<0.001				

^a^Adjusted for all variables in the column (age, sex, district, seeing, hearing, walking, cognition and self care).

## Discussion

Using a large sample of 12,841 individuals, we report that the prevalence of major depression is 4.5% in adult patients attending primary care settings in the Northern Province, four years after the end of a protracted 30-year armed conflict. Prevalence of mild depression is almost 3-fold higher in the study population (13.3%). Furthermore, the study reveals that older individuals, women and persons with disability are at a greater risk for depression.

To our knowledge, this is the first study that assessed depression within a population accessing primary care settings in Sri Lanka. The study design therefore yielded towards a public health approach to understand depression [34,35]. From the health services point-of-view, knowledge on burden of depression in a population that seeks care from primary care settings than in the general population would be more beneficial to provide targeted services. Further, the post-conflict nature of the study population provides a unique situation and critical evidence for policy and action.

It is noteworthy that the study finds higher prevalence of major depression (4.5%) in primary health care settings in the Northern province than the national estimate of 2.6% of major depression reported in 2007 [15]. This may be due to the fact that the national survey excluded conflict affected districts in the Northern province (due to inability of research teams to access those areas due to ongoing conflict), and that it was a community-based survey rather than a study among those seeking health care. Our estimate of major depression is somewhat closer to findings of a recent study which found major depression at 5.1% (95% CI: 3.2-7.7) in an internally displaced community due to war in the Northern province [22]. However, contextual difference and lower precision of the estimate in the latter study may limit the comparability of findings.

Depression can aggravate existing illnesses, signs and symptoms and vice-versa. Therefore, it is important to identify patients at high risk for depression so that these patients could be targeted for depression screening and treatment. Knowledge about risk factors or predictors of depression can ease identification of these patients. Regarding factors associated with depression, advancing age represents the strongest factor in our analysis, showing a linear trend with age. Although, an incremental increase of odds for depression with increasing age is observed in the univariable analysis, this stepwise pattern is not reflected in the multivariable analysis. Instead, the multivariable analysis revealed a greater but approximately equal odds (each age group showed an OR of around 5), for depression relative to the youngest age group of 18–24 years. This could be interpreted as younger age group being relatively at less risk to all other age groups in the context of other risk factors such as sex, and disabilities. Previous findings support the evidence that age contributes significantly to the prediction of depression [10,36,37]. While older adults may face widowhood, loss of function, or loss of independence, depression is not a ‘normal’ symptom of aging [38]. Studies show that depression that initially appears later in life is linked to a more chronic course of illness [39,40]. Living with untreated depression presents a serious public health problem since it may complicate chronic conditions such as heart disease, diabetes, and stroke; often accompanies functional impairment and disability and leads to increased health care costs [41,42]. Depression among older adults can be addressed through better community-based approaches for identifying and treating depression, and through more public awareness programmes [40,43,44].

Females are at a higher risk for depression than males; however the odds are marginal in our results. Many previous studies found a similar pattern [10,30], while some studies could not reveal significant sex differences [22,37]. The district differences of depression could be attributed to many external factors that were not included in the present study. There are differences across districts in the extent of re-settled population, livelihood development programmes, employment opportunities and access to health and other social services, that would affect the risk of depression [32]. Our study found a strong positive association between major depression and disabilities, however the cross-sectional nature of the study design limits conclusion whether disability led to depression or vice-versa. A cohort study of 6247 subjects 65 years and older in USA, who were initially free of disability has revealed that depression in elder patients caused limitation in activities of daily living and mobility after 6 years of follow-up [45]. This excess risk is partly explained by depressed persons’ decreased physical activity and social interaction. Further, there is evidence that improved depression by treatment reduces disability days and disability scores in depression persons [46]. Since depression and disabilities go hand-in-hand, further evidence through randomized trails would be needed to see effects of reduction of one on another.

Trauma and potential exposure to traumatic events due to protracted civil conflict appear to be associated with adverse mental health symptoms [47]. A study conducted among residents in Jaffna district in Sri Lanka in the aftermath of war revealed that the prevalence of symptoms of war-related mental health conditions was substantial and significantly associated with displacement status and underlying trauma exposure [21]. The same study found that approximately 68% of Jaffna residents experienced at least 1 trauma event and most individuals experienced multiple traumas. Furthermore, a dose–response relationship between the number of trauma events and psychiatric morbidity was evident, and chronic exposures to trauma events corresponded with higher levels of PTSD, anxiety, and depression symptoms. A qualitative inquiry into the psychosocial situation among internally displaced persons concluded that the collective trauma, i.e., traumatic psychological effect shared by community, can be profound [48]. However, the present study failed to collect data on the previous trauma exposure among individual participants. Despite, we can confirm that all communities which were serviced by the selected primary care facilities were affected by collective trauma.

The major implication of our findings on the health system is the importance of ensuring support to primary health care services for early detection and referral of common mental health conditions. Thus, training and sensitization of primary health care personnel at PMCU and DH can indeed make a difference for early detection of common mental health disorders, especially major depression. Capacity of the health personnel at primary care level should be enhanced in the area of mental health. These personnel should be trained to diagnose depression at primary health care level and to conduct initial basic management including counseling. It is also necessary to explore the referral system and continuity of care which were not addressed by the present study. Previous literature highlights that treatment gap and stigma are major barriers for communities to seek care at the primary health care settings for mental and psychological illnesses [49,50]. Therefore, further research is needed to address these issues such as treatment gap and stigma on mental illnesses. Since the prevalence of any form of depression including the mild forms, is high, we recommend that community-based mental health programmes be strengthened to increase knowledge and skills of community level workers to deal with common mental health and psychosocial issues and psychosocial problem solving. Our findings support the recommendations of a recent qualitative study aiming to rebuild family and community agency and resilience [24].

A number of limitations of our study has to be noted. First, though many studies demonstrate that the PHQ-9 has proven to be a sensitive and specific measure, the final diagnosis needs to be confirmed by a clinical assessment. Our study adopted a health systems perspective where the assessments occurred in routine primary care settings. Obtaining a definitive diagnosis for all those entering primary health care centres for treatment would be highly resource intensive, and warrant dedicated health programs from health system. Second, we did not cover all potential predictor variables due to the fact that the data collection was done during the history taking time of each patient at the out-patient department, consecutively on all patients. Our aim was to reveal the best determinants out of few easily obtainable parameters such as age, sex, occupation and disability etc. A major limitation of our study was the exclusion of the trauma scales. Due to the sensitive nature of the questions, administration of the trauma exposure scale was considered as ethically unsound and not approved by the health and administrative authorities of the Northern Province. Finally, this may not be representative of the population of the Northern Province, since the sample was obtained from primary health care facilities. Despite the mentioned weaknesses, hitherto this study is the largest study that has assessed depression among those seeking primary health care in Sri Lanka covering a large segment of the population. The quality of data was maintained at a high degree, especially with the PHQ-9 as described by Kroenke and Spitzer [26].

## Conclusion

In conclusion, this study reports that the prevalence of major depression is 4.5% (95% CI: 4.1-4.9) in adult patients attending primary care settings in the Northern Province, and that the older individuals, women and persons with disability are at a greater risk for depression. Prevalence of mild depression is almost 3-fold higher in the study population. The results indicate that the services at primary health care settings should be strengthened to screen patients for depression and advise accordingly. Strengthening community mental health services is necessary to detect psychological issues early and manage such issues promptly.

## Competing interests

The authors declare that they have no competing interests.

## Authors’ contributions

US participated in the design of the study, trained data collectors, performed the statistical analysis and drafted the manuscript. KW conceived of the study, and participated in its design, coordinated data collection and helped to draft the manuscript. SLP participated in the design of the study, coordination and supervision of data collection and helped to draft the manuscript. All authors read and approved the final manuscript.

## Pre-publication history

The pre-publication history for this paper can be accessed here:

http://www.biomedcentral.com/1471-244X/14/85/prepub
